# Learning leads to bounded rationality and the evolution of cognitive bias in public goods games

**DOI:** 10.1038/s41598-019-52781-7

**Published:** 2019-11-08

**Authors:** Olof Leimar, John M. McNamara

**Affiliations:** 1 Department of Zoology, Stockholm University, SE-106 91 Stockholm, Sweden; 2 School of Mathematics, University of Bristol, Bristol, BS8 1UG UK

**Keywords:** Behavioural ecology, Evolutionary theory, Psychology

## Abstract

In social interactions, including cooperation and conflict, individuals can adjust their behaviour over the shorter term through learning within a generation, and natural selection can change behaviour over the longer term of many generations. Here we investigate the evolution of cognitive bias by individuals investing into a project that delivers joint benefits. For members of a group that learn how much to invest using the costs and benefits they experience in repeated interactions, we show that overestimation of the cost of investing can evolve. The bias causes individuals to invest less into the project. Our explanation is that learning responds to immediate rather than longer-term rewards. There are thus cognitive limitations in learning, which can be seen as bounded rationality. Over a time horizon of several rounds of interaction, individuals respond to each other’s investments, for instance by partially compensating for another’s shortfall. However, learning individuals fail to strategically take into account that social partners respond in this way. Learning instead converges to a one-shot Nash equilibrium of a game with perceived rewards as payoffs. Evolution of bias can then compensate for the cognitive limitations of learning.

## Introduction

Many different cognitive processes fall under the heading of learning. The most basic is when an individual learns solely from rewards, without forming a more sophisticated cognitive model of the situation. This corresponds to the much-studied learning processes in classical and operant conditioning in animal psychology^1^, as well as to the standard, model-free approach to reinforcement learning in the study of machine learning^2^. It is this kind of learning we investigate here, where individuals explore through randomness in their actions and come to prefer actions that result in higher than so-far estimated rewards.

In social interactions, individuals typically vary in their characteristics in ways that influence costs and benefits. Examples could be differences in size and strength in aggressive interactions and variation in individual quality in cooperative interactions^3,4^. Variation in quality can cause individuals to vary in their investments into a joint project, which in turn can have the consequence that social partners respond through changes in their own investments. A question we raise is whether reinforcement learning allows individuals to take such dynamic responses from social partners into account when adjusting their own investments. As we show, the answer to the question can be no, because the responses by social partners occur over a too long time scale to be captured by learning. Instead, we show that the investment outcome of reinforcement learning in repeated rounds of the game corresponds to a Nash equilibrium of a one-shot game with the rewards acting as payoffs that are known to all players. Such a property of learning being myopic to future consequences of current actions can be seen as a kind of bounded rationality^5,6^. The phenomenon leaves open the possibility that evolutionary changes in the perceived rewards instead adjusts behaviour in a way that takes into account responses by social partners. The process can be thought of as an evolution of a bias in the innate perception of rewards, referred to as primary rewards or reinforcements in animal psychology. We show that such an evolution of cognitive bias indeed can occur, through the evolution of a tendency for individuals to act as if they underestimate their own quality, entailing an overestimation of their Darwinian fitness cost of investing into a project. The net effect is a lowering of investments compared to what would be the case for a Nash equilibrium of a one-shot game where individuals know the qualities of all players.

Our analysis is inspired by McNamara *et al*.^7^, who studied negotiation rules in games of cooperation with continuous actions. Our approach is to let reinforcement learning give rise to a “negotiation rule”, and then to examine the evolutionary consequences of such a rule. We study a public goods game where in each round each group member invests an amount into a joint project and shares equally in the benefit of the total investment by the group. Over the rounds, individuals learn to adjust their investments. For the learning dynamics, we use the actor-critic approach to reinforcement learning^2^, which is similar to so-called Bush-Mosteller learning^8^. We use a combination of analytical derivation and individual-based simulation to reach our main conclusion, that cognitive bias evolves as a consequence of the bounded rationality of learning. In summary, we show that if learning is driven by short-term rewards, cognitive biases may evolve as a compensating mechanism.

## Results

### Model overview

In each generation there are a number of investment rounds, *t* = 1, …, *T*, with an investment game involving a group of individuals. A group of size *g* stays together for life and *a*_*it*_ is the investment by individual *i* in round *t*. Each game is independent and has the same payoff structure, and group members can learn about the rewards (payoffs) from the successive rounds. Group members can differ in individual quality *q*_*i*_, which influences the cost of investment. The quality is a non-genetic aspect of an individual’s phenotype that influences its capacity to invest. The qualities are assumed not to vary between rounds of the game, but an individual’s quality is drawn randomly from a distribution at the start of a generation.

**Figure 1:**
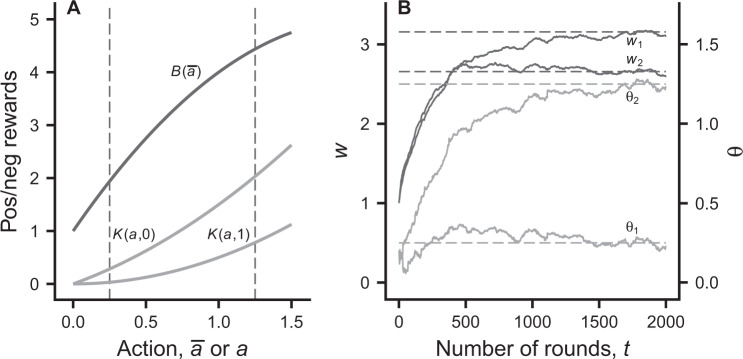
Illustration of the learning model. Panel (A) illustrates the benefit and cost as functions of the investment actions. The two curves for the cost correspond to qualities *q* = 0 and *q* = 1. See Eqs (2, 3) for the formulas. Panel (B) shows simulated learning dynamics of the estimated values *w*_*i*_ and mean actions *θ*_*i*_ for an interaction between two individuals with qualities *q*_1_ = 0 and *q*_2_ = 1. The dynamics of *w*_*i*_ and *θ*_*i*_ are given in Eqs (7, 10). The starting point of learning was (arbitrarily) chosen as *w*_*i*_ = 1.0 and *θ*_*i*_ = 0.2. The dashed lines are one-shot game predictions for the estimated value *w*_*i*_ and the mean investment *θ*_*i*_, corresponding to the investments in Eq. (14). These values of *θ*_*i*_ are also indicated in panel (A). Parameter values are: *g* = 2, *B*_0_ = 1, *B*_1_ = 4, *B*_2_ = −2, *K*_1_ = 1, *K*_11_ = 1, *K*_12_ = −1, *σ* = 0.05, *α*_*w*_ = 0.04, and *α*_*θ*_ = 0.002.

Concerning what is “known” by group members, we assume that they do not have any particular information, including about their own quality, but that they learn about which investment to make through the rewards they receive. We thus assume that at the start of a generation individuals do not have information about any of the *q*_*i*_ in the group, and during the interaction they perceive their own rewards. This situation corresponds to traditional instrumental or operant conditioning, but in a game situation. The net reward for individual *i* from round *t* is a benefit *B*, which depends on the group mean investment, minus a cost *K*, which depends on the individual’s own investment and quality (Fig. 1A and Eqs 1–4). We first assume that payoffs are perceived as rewards by the players. To study the evolution of cognitive bias, we than investigate whether individuals could evolve to perceive rewards that differ from the payoffs that correspond to Darwinian fitness.

### Actor-critic learning

We implement the repeated investment game as a reinforcement learning process, using the actor-critic method described in sections 13.5–13.7 of^2^, for the case without state transitions (only one state). Individuals learn which actions to use from the rewards they perceive. They use a temporal difference (TD) method to update a value *w*_*it*_ (estimated value by individual *i* at the start of round *t*), involving a TD error, or prediction error, which is the difference between actual and estimated rewards. The prediction error can be thought of as a reinforcement. Individuals select actions using a policy, expressed as a probability density *π*(*a*|*θ*_*it*_) of using the action *a*, assumed to be normal with mean *θ*_*it*_ and standard deviation (SD) *σ*. A so-called policy gradient method (ch. 13 in^2^) is used to update the parameter *θ*_*it*_, representing the mean investment action. In the learning process, the *w*_*it*_ and *θ*_*it*_, *i* = 1, …, *g*, then perform a random walk in a 2*g*-dimensional space (Fig. 1B), specified by Eqs 5–11; (see Methods).

**Figure 2:**
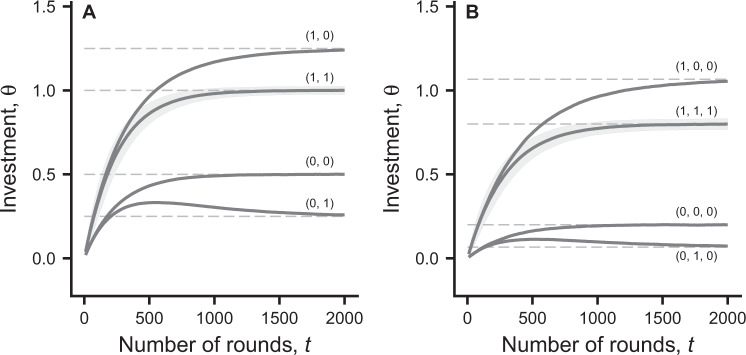
Mean and SD of simulated investment actions for individual *i* = 1 in populations of groups, plotted over the rounds of learning. At the start of learning, individuals are assigned random qualities from the set {0, 1} and the curves are labelled with with the qualities, *q*_*i*_, *i* = 1, …, *g*, of individuals in a group. The spread (SD) of values of *θ* in the population is shown as grey shading only for the subset of groups where all *q*_*i*_ = 1 (for clarity, to avoid overlap). Panel (A) shows all cases of group compositions with *g* = 2, namely groups with *q*_1_ = 1, *q*_2_ = 1; *q*_1_ = 1, *q*_2_ = 0; *q*_1_ = 0, *q*_2_ = 1; and *q*_1_ = 0, *q*_2_ = 0. Panel (B) shows a subset of cases of group compositions with *g* = 3, labelled *q*_1_, *q*_2_, *q*_3_. The total population size is 24 000 individuals in both panels. The dashed lines are one-shot game predictions, from Eq. (14). Other parameters are as in Fig. 1.

Reinforcement learning based on a policy gradient is thought to have good convergence properties (e.g., ch. 13 in^2^), in the sense that for small rates of learning a local optimum is approached. In a game situation, the outcome of learning in successive rounds might approximate a Nash equilibrium of a one-shot game with the rewards as payoffs. In this one-shot game the payoffs, including the dependence on individual qualities, are known to the players, and are given by Eq. (4). From our individual-based simulations (Figs 1B and 2), the learning dynamics approach this Nash equilibrium, which is specified by Eqs (13, 14). Because learning is a stochastic process, driven by the individual exploratory choices of investment actions, there is variation in learning trajectories between groups with identical compositions of qualities. This variation is shown as shading, indicating ±1 SD, in Fig. 2. For small rates of learning we also show that the learning dynamics is approximately a vector autoregressive process^9^ around the Nash equilibrium (see SI, Figs S1 and S2).

### Evolution of cognitive bias

We found that the learning outcome corresponds to a Nash equilibrium of a one-shot game, with payoffs illustrated in Fig. 1A and specified in Eq. (4). For these payoffs, the cost of an action depends on the “true” quality *q*_*i*_ of a player. However, the analysis of learning applies in the same way if the qualities *q*_*i*_ are replaced by “perceived qualities” *p*_*i*_, as in Eq. (15), meaning that individual *i* behaves as if its quality is *p*_*i*_. We refer to the rewards used in learning as “perceived rewards”. An individual of quality *q*_*i*_ would then learn from rewards corresponding to its perceived quality *p*_*i*_, which might differ from *q*_*i*_. Note that we assume that individuals only perceive their benefit and cost in each round. The bias thus occurs in an individual’s perception of its cost of investment, but for convenience we express it as a bias in perceived quality. Specifically, an individual is assumed to perceive a cost that corresponds to its perceived quality *p*_*i*_, while its Darwinian fitness cost is given by its true quality *q*_*i*_. We define an individual’s cognitive bias as the difference between its perceived and true qualities: *d*_*i*_ = *p*_*i*_ − *q*_*i*_. We also assume that perceived qualities satisfy *p*_*i*_ ≤ 1 and can be negative. This allows *d*_*i*_ to be either positive or negative, with negative *d*_*i*_ corresponding to higher perceived costs of investment.

**Figure 3:**
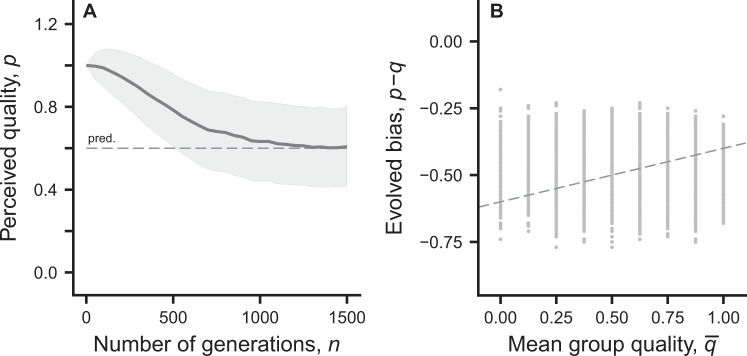
Illustration of the evolution of individual perceived quality *p*_*i*_, through the genetically determined cognitive bias *d*_*i*_ = *p*_*i*_ − *q*_*i*_, from individual-based simulation of populations similar to those illustrated in Fig. 2. Panel (A) shows evolution of mean and SD of *p*_*i*_ = *q*_*i*_ + *d*_*i*_ over the generations in a population with groups of size *g* = 2, with true qualities *q*_1_ = 1, *q*_2_ = 1 (i.e., all individuals have true quality 1). The mutation rate for alleles for *d*_*i*_ is 0.05 and the mutant increment is normally distributed with an SD of 0.04. The dashed line is the prediction from Eq. (17). Panel (B) shows the bias *d*_*i*_, as a function of the mean quality $\bar{q}$ in the group, for a population with groups of size *g* = 2 and with true qualities selected randomly at the start of a generation from the set {0.00, 0.25, 0.50, 0.75, 1.00}. The dashed line shows the prediction from Eq. (17) for each group composition in the final simulated generation. The mutation rate per allele for *d*_*i*_ is 0.001 with SD of mutant increments of 0.04. Other parameters are as in Fig. 1.

A main result of our analysis is that when there are social partners, i.e. for group size *g* > 1, zero cognitive bias, i.e. *d*_*i*_ = *p*_*i*_ − *q*_*i*_ = 0, is not an evolutionary equilibrium, but instead a negative bias evolves. An intuitive explanation is that, given the perceived qualities of the group members, learning approaches a one-shot Nash equilibrium for these perceived qualities. The learning outcome does not strategically take into account that social partners respond to an individual’s lowered investment by increasing their investments somewhat. From the definition of a Nash equilibrium, it then follows that the individual can gain fitness by having a cognitive bias, i.e., by lowering its perceived quality from *p*_*i*_ = *q*_*i*_. In effect, an individual whose perceived quality is lower than the real quality makes smaller investments, which in turn means that other players end up making larger investments. The individual thus makes a fitness gain from the biased perception. The derivation of this result appears in the Methods, Eq. (16), and the evolutionarily equilibrium bias is given in Eq. (17), with detailed derivation in SI. This result is illustrated in Fig. 3A, which shows the evolution of a genetically determined perceived quality *p*_*i*_ in a population where all individuals have true quality *q*_*i*_ = 1. As can be seen, a negative cognitive bias *d*_*i*_ = *p*_*i*_ − *q*_*i*_ evolves.

For a given composition of *q*_*i*_, *i* = 1, …, *g*, in a group one can find the evolutionarily stable perceived qualities ${p}_{i}^{\ast }$ using Eq. (16). For the benefit and cost functions in Eqs (2, 3), which we use for illustration, this simplifies to Eq. (17), from which it follows that the evolutionarily stable cognitive bias ${d}_{i}^{\ast }={p}_{i}^{\ast }-{q}_{i}$ depends on the group average quality $\bar{q}$. However, it is not reasonable to assume that an individual has an evolved innate underestimation of its true quality that depends on the particular group composition, because this composition is not known to the individual at the start of a generation. Instead, in individual-based simulations we assume that the trait that evolves is simply a bias *d*_*i*_, such that the perceived quality is *p*_*i*_ = *q*_*i*_ + *d*_*i*_, irrespective of the kind of group the individual is a member of. An example with *g* = 2 and variation in true quality in the population appears in Fig. 3B. Our assumption means that perceived qualities cannot match the prediction from Eq. (17) for each particular group composition (Fig. 3B), but there is agreement between the population averages of the evolved and predicted cognitive biases (equal to −0.49 and −0.50, respectively).

**Figure 4:**
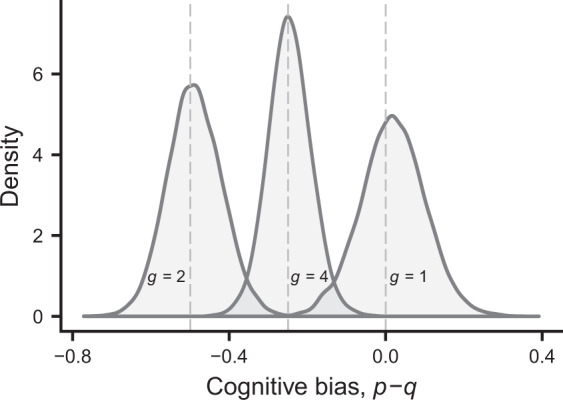
Illustration of the distribution of evolved cognitive bias *d*_*i*_ = *p*_*i*_ − *q*_*i*_ for different cases of group sizes. Parameters are as in Fig. 3B, and the distribution for *g* = 2 comes from the population illustrated in Fig. 3B. The dashed lines give the prediction from Eq. (17), averaged over the different group compositions in the population.

This is further illustrated in Fig. 4, showing the outcome of individual-based simulations for populations with different group sizes. The most extreme bias occurs for *g* = 2, and as the group size *g* becomes large, the bias approaches zero (see SI). For solitary investing individuals (*g* = 1), there is no bias on average.

## Discussion

A major conclusion from our analysis is that when individuals in a group learn how much to invest in a public goods game, there is scope for the evolution of cognitive bias, corresponding to an evolution of the perceived cost of investment into the public good (Figs 3 and 4). The reason is that cognitive limitations of reinforcement learning prevent individuals from fully taking into account how social partners respond to variation in the ability of individuals to invest. Reinforcement learning is a mechanism driven by immediate rewards, without foresight about the medium-term outcome of learning. This aspect of learning can be seen in Figs 1B and 2, where the mean investment *θ* of the lowest-quality individual in a group approaches its equilibrium by first overshooting the eventual equilibrium value. Furthermore, the learning interaction is particularly beneficial for a low-quality individual (Fig. 1B), who ends up investing little, when interacting with higher-quality partners who end up investing more, by learning to compensate for the shortfall. This explains why evolutionary changes are in the direction of a reduced perceived quality, i.e. a negative cognitive bias.

For an individual to learn about how social partners respond to variation in its tendency to invest, several interactions with different social groups would be needed, where the individual could explore the consequences of changes in its tendency to invest. Even so, for an individual to learn that lowering its current investment increases rewards in future rounds, because others learn to increase their investments, the individual must connect current behaviour to future rewards. Animal psychology has shown that this can be difficult to do, in particular without any indicators to the individual that there might be such a causal connection. Pavlov^10^ discovered that the time interval between conditioned and unconditioned stimuli (the CS-US interval) needs to be short for an association to be formed. Exceptions to this rule represent special adaptations, of which taste aversion learning is the best known^1^. It has also been shown that learning can occur for longer CS-US intervals, if the CS is highly salient and there are no interfering stimuli during the interval^1^. A clearly perceived chain of states and actions, leading to a goal, can also support more sophisticated learning about future consequences of current actions^11^, but learning about social partners does not have a structure of that kind. It thus seems reasonable that unless individuals have some other special preparedness to connect current behaviour to medium-term rewards, mediated through the responses of social parters, this will be difficult to learn.

### Game dynamics and learning

As described by Weibull in the proceeding from a Nobel seminar^12^, the general idea that players of a game are members of populations and revise their strategies in a more-or-less myopic fashion was introduced in unpublished work by John Nash. This is now a foundation for game theory in economics^13–15^, and has also been used for game theory in biology^16–18^. Game dynamics based on reinforcement learning, including the actor-critic method^2^, can be seen as a variant of this approach, with its learning mechanisms inspired by experimental psychology and neuroscience. Thus, the TD updating of an estimated value^2^, described in Eqs (6, 7), represent the critic component of an actor-critic mechanism and is connected to the influential Rescorla-Wagner model of classical conditioning^19^ as well as to the reward prediction error hypothesis of dopaminergic neuron activity^20^. For the actor component, from Eqs (10, 11), changes in the tendencies to perform actions depend on the covariance of eligibility and reward. This learning mechanism has been given an interpretation in terms of synaptic neural plasticity^2,21^. It is worth noting that there is a certain similarity between the actor-critic learning dynamics in Eq. (11) and the so-called Price equation for selection dynamics^22^. Although these equations describe fundamentally different processes, natural selection vs. actor-critic learning, they are both helpful in providing intuitive understanding.

### Bounded rationality

The cognitive limitations of learning have been put forward as an important reason for bounded rationality^6,23,24^ and our work gives further support to the idea. It is a general principle that certain aspects of the situation an individual finds itself in might be learnt very slowly or not at all, even though they could influence payoffs. In our model, the effects on rewards of responses of social partners, resulting from learning about an individual’s characteristics, do not influence the learning of investment actions. Instead, we found a learning outcome where investments converged on a Nash equilibrium of a one-shot game with perceived rewards as payoffs, even though group members stayed together over successive investment rounds and, in principle, might have discovered how social partners learn about investment variation.

### Evolution of cognitive bias

The possibility of cognitive bias in decision making has been of interest in economics, psychology and biology. Among the examples are the base rate bias^25^ and the judgement bias^26^. The general question of how to formulate an evolutionary theory of cognitive bias has also been raised^27^.

An insight from our analysis is that the bounded rationality of learning leaves scope for evolution to adjust the rewards (primary rewards or preferences) in a way that corresponds to a cognitive bias in an individual’s perception of its quality. With such a bias, learning by individuals results in an approach towards evolutionarily optimal behaviour. Our result is related to the idea of an “indirect evolutionary approach” in economic game theory^28,29^, where players are assumed to know or learn about each other’s preferences and to play a Nash equilibrium given the preferences, which are then assumed to be shaped by evolution. The connection with our work is that we showed that learning causes the investments to approach a one-shot Nash equilibrium given the perceived qualities, and the indirect evolutionary approach assumes that players know or find out each other’s preferences and play a Nash equilibrium given these preferences.

A widespread and successful idea in animal psychology is that evolution causes primary rewards to indicate Darwinian fitness^1^. More generally, it is a basic element of evolutionary biology and behavioural ecology that actions can be given a Darwinian currency, in the form of reproductive value^30,31^. Our work here, as well as related work in economic game theory^29,32,33^, shows that an exact correspondence between primary rewards and reproductive value need not hold. In our model this happened because of cognitive limitations of learning, although reproductive value was still important for the analysis.

As illustrated in Figs 3 and 4, there is variation between individuals in their cognitive bias, i.e. in how much their perceived qualities deviate from the true qualities, which is a consequence of a balance between selection, mutation and genetic drift. This is reminiscent of animal personality variation^34^, where individuals differ in important behavioural characteristics. One often assumes that disruptive selection lies behind personality variation^35^, but our results here show that there can be substantial variation also with stabilising selection on the trait in question. In general, whether selection is stabilising or disruptive, we propose that bounded rationality, from cognitive limitations of learning, opens up a possibility for individuals to vary in their characteristics, including cognitive biases in social interactions.

## Methods

### Model details

In round *t*, the group mean investment ${\bar{a}}_{t}$ is

1 $${\bar{a}}_{t}=\frac{1}{g}\mathop{\sum }\limits_{i=1}^{g}\,{a}_{it}.$$

The benefit of investment for each group member is assumed to be a concave, smooth function of the group average investment $\bar{a}$, having a negative second derivative. For illustration we use the special case

2 $$B(\bar{a})={B}_{0}+{B}_{1}\bar{a}+\frac{1}{2}{B}_{2}{\bar{a}}^{2},$$

where *B*_1_ > 0 and *B*_2_ < 0 (Fig. 1A). Maximum benefit occurs for $\bar{a}=-\,{B}_{1}/{B}_{2}$, and we might constrain actions to be smaller than this, to ensure that benefits increase with the actions. The cost *K*(*a*_*i*_, *q*_*i*_) of investment *a*_*i*_ by group member *i* is assumed to be a smooth convex and increasing function of *a*_*i*_ that increases more rapidly with *a*_*i*_ for smaller *q*_*i*_, and has a positive second derivative with respect to *a*_*i*_. For illustration we use

3 $$K({a}_{i},{q}_{i})={K}_{1}{a}_{i}+\frac{1}{2}{K}_{11}{a}_{i}^{2}+{K}_{12}{a}_{i}{q}_{i},$$

with *K*_1_ > 0, *K*_11_ > 0 and *K*_12_ < 0 (Fig. 1A). We thus have a public goods game in each round with the payoff to player *i* given by

4 $${W}_{i}({a}_{i},{a}_{-i},{q}_{i})=B(\bar{a})-K({a}_{i},{q}_{i}),$$

where *a*_−*i*_ denotes the vector of actions of all individuals in the group except for *i*.

### Reinforcement learning: the actor-critic approach

Actions are independent and normally distributed with mean *θ*_*it*_ and SD *σ*:

5 $$\pi (a|{\theta }_{it})=\frac{1}{\sqrt{2\pi {\sigma }^{2}}}\,\exp \,(-\frac{{(a-{\theta }_{it})}^{2}}{2{\sigma }^{2}}).$$

For simplicity, we keep *σ* constant and rather small, but we note that variation in *a* is needed for a learner to explore and thus to discover how actions can be improved. Keeping with reinforcement learning notational conventions, the reward from Eq. (4) for individual *i* from the play in round *t* is denoted *R*_*i*,*t*+1_. The TD error is given by

6 $${\delta }_{it}={R}_{i,t+1}-{w}_{it}={W}_{i}({a}_{it},{a}_{-it},{q}_{i})-{w}_{it}.$$

This is used to update the learning parameter *w*_*it*_ as follows:

7 $${w}_{i,t+1}={w}_{it}+{\alpha }_{w}{\delta }_{it},$$

where *α*_*w*_ is a learning rate parameter (we do not use discounting in our formulation of learning and each round is treated as a new episode^2^). The expected change in *w*_*it*_ is

8 $${\rm{E}}\,[{w}_{i,t+1}-{w}_{it}|{w}_{\cdot t},{\theta }_{\cdot t}]={\alpha }_{w}{\rm{E}}\,[{\delta }_{it}|{w}_{\cdot t},{\theta }_{\cdot t}].$$

For the actor-critic method the learning updates for the policy involve the derivative of the logarithm of *π*(*a*|*θ*) with respect to *θ*, given by

9 $${\zeta }_{it}=\frac{\partial \,\log \,\pi (a|{\theta }_{it})}{\partial {\theta }_{it}}=\frac{a-{\theta }_{it}}{{\sigma }^{2}},$$

which sometimes is referred to as an eligibility. The update to the learning parameter *θ*_*it*_ is

10 $${\theta }_{i,t+1}={\theta }_{it}+{\alpha }_{\theta }{\delta }_{it}\frac{\partial \,\log \,\pi (a|{\theta }_{it})}{\partial {\theta }_{it}}={\theta }_{it}+{\alpha }_{\theta }{\delta }_{it}{\zeta }_{it},$$

where *α*_*θ*_ is a learning rate parameter. It is worth noting that the expectation of the increment in *θ*_*i*_ is proportional to the covariance of the TD error and the eligibility:

11 $${\rm{E}}\,[{\theta }_{i,t+1}-{\theta }_{it}|{w}_{\cdot t},{\theta }_{\cdot t}]={\alpha }_{\theta }\,{\rm{Cov}}\,[{\delta }_{it},{\zeta }_{it}|{w}_{\cdot t},{\theta }_{\cdot t}].$$

A frequent issue for actor-critic reinforcement learning is how the learning rates *α*_*w*_ and *α*_*θ*_ should be chosen. Learning involves changes in both the estimated value *w*_*i*_ and the action mean value *θ*_*i*_ and both are driven by the TD error *δ*_*i*_. From Eqs (7, 10), noting that *a*_*i*_ − *θ*_*i*_ has a magnitude of about *σ*, we ought then to have

12 $${\alpha }_{w}\Delta \theta \sim \frac{{\alpha }_{\theta }}{\sigma }\Delta w$$

for learning to cause the *w*_*i*_ and *θ*_*i*_ to move over approximate ranges Δ*w* and Δ*θ*. We have used this relation in our learning simulations, with ranges Δ*w* and Δ*θ* of around 1.

Intuitively, from Eqs (6–10) we might expect ∂*W*_*i*_/∂*a*_*i*_ = 0 to hold approximately for a learning equilibrium. This would correspond to a Nash equilibrium, and is the motivation for the following analysis.

### One-shot game

By our assumptions about the payoffs, this is a concave game, and using a result in^36^ one can show that the game has a unique Nash equilibrium (see SI). This equilibrium should satisfy ∂*W*_*i*_/∂*a*_*i*_ = 0 or, from Eq. (4),

13 $$\frac{1}{g}B{\prime} ({\bar{a}}^{\ast })=\frac{\partial K({a}_{i}^{\ast },{q}_{i})}{\partial {a}_{i}},$$

for *i* = 1, …, *g*. It follows that $\partial K({a}_{i}^{\ast },{q}_{i})/\partial {a}_{i}=\partial K({a}_{j}^{\ast },{q}_{j})/\partial {a}_{j}$ and, because $\partial K({a}_{i}^{\ast },{q}_{i})/\partial {a}_{i}$ is increasing in *a*_*i*_ and decreasing in *q*_*i*_, that ${a}_{i}^{\ast } > {a}_{j}^{\ast }$ when *q*_*i*_ > *q*_*j*_, so higher-quality individuals invest more at the equilibrium. Furthermore, using results in^37^, one can show that ${a}_{i}^{\ast }$ increases with *q*_*i*_ and decreases with *q*_*j*_, *j* ≠ *i* (see SI, Equation S11). For our special case of Eqs (2, 3), one readily finds that

14 $${a}_{i}^{\ast }={e}_{0}+{e}_{1}{q}_{i}+{e}_{2}\sum _{j\ne i}\,{q}_{j}={e}_{0}+{e}_{1}{q}_{i}+{e}_{2}(g-1){\bar{q}}_{-i},$$

where, for *g* > 1, ${\bar{q}}_{-i}$ is the average quality of all individuals the group except for *i* (see SI, Equation S21, for the coefficients). For large *g* we see from Eq. (13) that the equilibrium is for individual *i* to minimize *K*(*a*_*i*_, *q*_*i*_).

### Evolution of cognitive bias

The cost *K*(*a*_*i*_, *q*_*i*_), from Eq. (3), is assumed to be the true cost of investment, measured in terms of Darwinian reproductive value, for an individual with true quality *q*_*i*_. We also assume that $B(\bar{a})$, from Eq. (2), corresponds to reproductive value. These reproductive values represent payoffs in the standard sense of evolutionary game theory. The meaning of the perceived quality *p*_*i*_ is that the individual perceives the cost *K*(*a*_*i*_, *p*_*i*_), in the sense of rewards influencing learning.

Let ${a}_{i}^{\ast }({p}_{\cdot })$ be a Nash equilibrium where the true qualities in Eq. (13) are replaced by perceived qualities, thus satisfying

15 $$\frac{1}{g}{B}^{{\rm{{\prime} }}}({\bar{a}}^{\ast }({p}_{\cdot }))=\frac{{\rm{\partial }}K}{{\rm{\partial }}{a}_{i}}({a}_{i}^{\ast }({p}_{\cdot }),{p}_{i}),$$

for *i* = 1, …, *g*. If the true qualities of group members are *q*_*i*_, an evolutionary equilibrium for the perceived qualities *p*_*i*_ should satisfy (see SI)

16 $$\begin{array}{ccc}\frac{d{W}_{i}}{d{p}_{i}} & = & [\frac{{\rm{\partial }}K}{{\rm{\partial }}{a}_{i}}({a}_{i}^{\ast }({p}_{\cdot }),{p}_{i})-\frac{{\rm{\partial }}K}{{\rm{\partial }}{a}_{i}}({a}_{i}^{\ast }({p}_{\cdot }),{q}_{i})]\frac{{\rm{\partial }}{a}_{i}^{\ast }({p}_{\cdot })}{{\rm{\partial }}{p}_{i}}\\  &  & +\,\frac{1}{g}{B}^{{\rm{{\prime} }}}({\bar{a}}^{\ast }({p}_{\cdot }))\sum _{j\ne i}\frac{{\rm{\partial }}{a}_{j}^{\ast }({p}_{\cdot })}{{\rm{\partial }}{p}_{i}}=0.\end{array}$$

From this it follows that *p*_*i*_ = *q*_*i*_ is not an evolutionary equilibrium for *g* > 1, because the expression in the square bracket is then zero and the other term is negative, because $\partial {a}_{j}^{\ast }/\partial {p}_{i} < 0$ for *j* ≠ *i*. This shows that an individual could gain fitness by lowering its perceived quality from *p*_*i*_ = *q*_*i*_ to *p*_*i*_ = *q*_*i*_ + *d*_*i*_ with *d*_*i*_ < 0.

In such a case, an individual with true quality *q*_*i*_ will perceive the cost *K*(*a*_*i*_, *p*_*i*_) = *K*(*a*_*i*_, *q*_*i*_ + *d*_*i*_). For our special case of Eq. (3), this means that the individual perceives an extra cost, or penalty, *K*_12_*d*_*i*_*a*_*i*_ of the investment *a*_*i*_. The solution to Eq. (16) for the special case can be written as

17 $${p}_{i}^{\ast }-{q}_{i}={\beta }_{0}+{\beta }_{1}\bar{q},$$

which is worked out in the SI, with *β*_0_ and *β*_1_ given in Equation (S30). For *g* = 1 one sees from Equation (S30) that *β*_0_ = *β*_1_ = 0, so that ${p}_{1}^{\ast }={q}_{1}$ is the solution.

### Individual-based simulations

For individual-based simulation of the actor-critic learning dynamics, we constructed populations of individuals, each with a randomly assigned quality, split into groups of size *g*. For ease of interpretation, qualities were drawn from a small set of values for *q*, for instance *q*_*i*_ ∈ {0, 1} in Fig. 2. In this population, the learning dynamics follows Eqs (5–10) over rounds *t* = 1, …, *T*. The aim of the simulations is to compare the outcome of learning with the one-shot Nash equilibrium predictions from Eq. (14). For evolutionary simulations, over many generations, we implemented discrete, non-overlapping generations and assumed individuals to be hermaphrodites with one diploid locus additively determining the trait *d*_*i*_ = *p*_*i*_ − *q*_*i*_. The time sequence of events for evolutionary simulations was as follows: (*i*) random sorting of newborn individuals into groups and assignment of random true qualities; (*ii*) learning dynamics over *T* rounds, with the perceived quality of an individual given as

18 $${p}_{i}={q}_{i}+{d}_{i},$$

where *d*_*i*_ is the individual’s genetically determined trait; (*iii*) assignment of a Darwinian payoff to each individual, computed as the individual’s average payoff over the rounds, based on its true quality; and (*iv*) formation of the next generation through mating, including mutation, with the probability of being chosen as parent being proportional to an individual’s payoff.

## Supplementary information

Supplementary information for “Learning leads to bounded rationality and the evolution of cognitive bias in public goods games”

## Data Availability

Source code for the individual-based simulations is available at GitHub, together with instruction for compilation on a Linux operating system, and with example input files: https://github.com/oleimar/pggsim. The R code and individual-based simulation output used to generate the figures are available from the corresponding author on reasonable request.
