# Supplementary material for: Learning leads to bounded rationality and the evolution of cognitive bias in public goods games
Source: Sci Rep. 2019 Nov 8;9:16319. doi: 10.1038/s41598-019-52781-7 (PMC6841956; doi:10.1038/s41598-019-52781-7)
Supplement: Supplementary file 1 — Supplementary information for “Learning leads to bounded rationality and the evolution of cognitive bias in public goods games” [file 41598_2019_52781_MOESM1_ESM.pdf]

Supplementary information for "Learning leads to bounded rationality and the evolution of cognitive bias in public goods games"

Olof Leimar and John M. McNamara

Numbers in square brackets refer to the list of references and those in round brackets to equations, both in the main text.

Payoff function and one-shot game

For the payoff function in equation (4), we note that

$$\frac{\partial W_i}{\partial a_j} = \frac{1}{g} B'(\bar{a}) - \frac{\partial K(a_i, q_i)}{\partial a_i} \delta_{ij} \tag{S1}$$

and

$$\frac{\partial^2 W_i}{\partial a_j \partial a_k} = \frac{1}{g^2} B''(\bar{a}) - \frac{\partial^2 K(a_i, q_i)}{\partial a_i^2} \delta_{ij} \delta_{ik}, \tag{S2}$$

where δ_{ij} is the Kronecker delta, which is equal to one if $i = j$ and zero otherwise. By our assumptions about the payoffs, the matrix with elements given by equation (S2) for $i = 1, \ldots, g$, $j = i$, $k = 1, \ldots, g$ is symmetric and negative definite. According to [36], this implies that a one-shot game with this payoff function has a unique Nash equilibrium.

Approximate actor-critic process

Here we investigate the actor-critic learning dynamics in the vicinity of a one-shot Nash equilibrium, for low rates of learning and small σ, and assuming that $g > 1$. With $a_i^* = a_i^*(q_\cdot)$ the equilibrium from equation (13), i.e. (14, S21) for our special case of equations (2, 3), we define the deviations

$$\begin{aligned} x_{it} &= w_{it} - W_i^* \\ y_{it} &= \theta_{it} - a_i^* \\ z_{it} &= a_{it} - \theta_{it} = a_{it} - (a_i^* + y_{it}), \end{aligned}$$

where $W_i^* = W_i(a_i^*, a_{-i}^*, q_i)$. Because $\partial W_i(a_i^*, a_{-i}^*, q_i)/\partial a_i = 0$ at the Nash equilibrium, we have the Taylor expansion

$$\begin{aligned} R_{it} =& W_i(a_i^* + y_{it} + z_{it}, a_{-i}^* + y_{-it} + z_{-it}, q_i) \\ =& W_i^* + \sum_{j \neq i} \frac{\partial W_i(a_i^*, a_{-i}^*, q_i)}{\partial a_j} \left(y_{jt} + z_{jt}\right) + \\ & \frac{1}{2} \sum_{j=1}^{g} \sum_{k=1}^{g} \frac{\partial^2 W_i(a_i^*, a_{-i}^*, q_i)}{\partial a_j \partial a_k} \left(y_{jt} + z_{jt}\right)\left(y_{kt} + z_{kt}\right) + \cdots \\ =& W_i^* + \omega_1 \sum_{j \neq i} \left(y_{jt} + z_{jt}\right) + \omega_{11} \sum_j y_{jt} z_{it} + \omega_{22} y_{it} z_{it} + \cdots. \end{aligned} \tag{S3}$$

For convenience we used the notation

$$\begin{aligned} \omega_1 &= \frac{1}{g} B'(\bar{a}^*) = \frac{1}{g}\left(B_1 + B_2 \bar{a}^*\right) \\ \omega_{11} &= \frac{1}{g^2} B''(\bar{a}^*) = \frac{1}{g^2} B_2 \\ \omega_{22} &= -\frac{\partial^2 K(a_i^*, q_i)}{\partial a_i^2} = -K_{11}, \end{aligned}$$

where the expressions on the right-hand side are for the special case. The reason for writing out second order terms like $y_{jt} z_{it}$ in equation (S3) is that they contribute to the covariance below. Because the z_{jt} are (independent and) normal with mean zero and

standard deviation σ, the expectation of the TD error in equation (8), conditional on the x_{jt} and y_{jt}, $j = 1,\ldots,g$, is

$$\begin{aligned}
\mathrm{E}\big[\delta_{it}|x_{\cdot t}, y_{\cdot t}\big] &= \mathrm{E}\big[R_{it}|x_{\cdot t}, y_{\cdot t}\big] - (W_i^* + x_{it}) \\
&= -x_{it} + \omega_1 \sum_{j\neq i} y_{jt} + \cdots,
\end{aligned} \tag{S4}$$

which gives the deterministic part of the increment in equation (7). For equations (10, 11), we need the (conditional) covariance of δ_{it} with the eligibility ζ_{it} in order to compute the deterministic part of the increment in the learning parameter θ_{it}. We get the covariance

$$\begin{aligned}
\mathrm{Cov}\big[\delta_{it}, \frac{a_{it}-\theta_{it}}{\sigma^2}|x_{\cdot t}, y_{\cdot t}\big] &= \frac{1}{\sigma^2}\mathrm{E}\left[\Big(\sum_j \omega_{11} y_{jt} + \omega_{22} y_{it}\Big) z_{it}^2 \Big| x_{\cdot t}, y_{\cdot t}\right] + \cdots \\
&= \sum_{j\neq i} \omega_{11} y_{jt} + (\omega_{11} + \omega_{22}) y_{it} + \cdots.
\end{aligned} \tag{S5}$$

Because the eligibility from equation (9) is equal to z_{it}/σ^2, which has variance $1/\sigma^2$, becoming big for small σ, terms containing z_{it}, like $\omega_{11} y_{it} z_{it}$ in equation (S3), contribute to lowest order to the covariance.

To approximate the actor-critic learning dynamics as a vector autoregressive process, we introduce the vector $\xi_t = (x_{1t},\ldots,x_{gt},y_{1t},\ldots,y_{gt})^T$, with elements $\xi_{lt}, l = 1,\ldots,2g$ (and if we approximate time to be continuous, we obtain a multivariate Ornstein-Uhlenbeck process). We then have a VAR(1) process given by

$$\xi_{t+1} = A\xi_t + \varepsilon_t, \tag{S6}$$

where the matrix A is expressed using the approximate deterministic dynamics, given in equation (S10) below, and ε_t is a vector of zero-mean, serially uncorrelated stochastic increments. The process is stable (stationary and ergodic) if the eigenvalues of A have modulus less than one (see a textbook on multivariate time series analysis, e.g., [9]. From equations (7, 10, S3), the stochastic increments are given by

$$\begin{aligned}
\varepsilon_{it} &= \alpha_w \omega_1 \sum_{j\neq i} z_{jt} + \cdots \\
\varepsilon_{g+i,t} &= \alpha_\theta \omega_1 \sum_{j\neq i} z_{jt} \frac{z_{it}}{\sigma^2} + \cdots
\end{aligned} \tag{S7}$$

for $i = 1,\ldots,g$. Except for the special case $g = 1$, which we do not consider here, the first of these, involving z_{jt}, is normally distributed to lowest order, but the second, involving products $z_{jt} z_{it}$ of two independent normally distributed variables, has a leptokurtic distribution. Nevertheless, for $g > 1$, numerical simulation of the learning dynamics shows that the equilibrium distribution of the process is approximately multivariate normal. Let P be the variance-covariance matrix of the increment vector ε_t to lowest order, which is given in equation (S11) below. The equilibrium variance-covariance matrix Q of the process ξ_t satisfies

$$Q = AQA^T + P, \tag{S8}$$

which is sometimes called the discrete-time Lyapunov equation. This is readily solved, as the linear system in equation (S12) below, or numerically through iteration. The solution Q was used to generate the comparison in Fig. S1, showing that the approximation is at least reasonable for low rates of learning, which is also illustrated in Fig. S2. We write the matrix A in equation (S6) as a block matrix

$$A = \begin{pmatrix} A_{11} & A_{12} \\ A_{21} & A_{22} \end{pmatrix}, \tag{S9}$$

with $g \times g$ matrices as blocks. From equations (7, 10, S4, S5), the blocks are given by

$$\begin{aligned}
A_{11} &= (1-\alpha_w) I_g \\
A_{12} &= \alpha_w \omega_1 (1 - I_g) \\
A_{21} &= 0 \\
A_{22} &= I_g + \alpha_\theta (\omega_{11} 1 + \omega_{22} I_g),
\end{aligned} \tag{S10}$$

where I_g is the $g \times g$ identity matrix and 1 and 0 in the last three equations indicate $g \times g$ matrices with all elements 1 and 0, respectively. The variance-covariance matrix P of the stochastic increments in equation (S7) is also expressed as a block matrix

$$\begin{aligned} P_{11} &= \alpha_w^2 \omega_1^2 \sigma^2 \big((g-2)1 + I_g\big) \\ P_{12} &= 0 \\ P_{21} &= 0 \\ P_{22} &= \alpha_\theta^2 \omega_1^2 \big((g-2)I_g + 1\big), \end{aligned} \tag{S11}$$

where 0 and 1 in the different equations indicate $g \times g$ matrices with all elements 0 and 1, respectively. Using the vectorization operator and the Kronecker product, equation (S8) can be written

$$\big(I_{4g^2} - A \otimes A\big)\,\mathrm{vec}(Q) = \mathrm{vec}(P). \tag{S12}$$

For a stable process, the $4g^2 \times 4g^2$ matrix $I_{4g^2} - A \otimes A$ can be inverted, providing the solution Q from $\big(I_{4g^2} - A \otimes A\big)^{-1}\,\mathrm{vec}(P)$.

Comparative statics of the Nash equilibrium

Here we show that, for a Nash equilibrium $a_i^*(q_\cdot)$, $i = 1, \ldots, g$, satisfying equation (13), the equilibrium actions depend on the qualities in the following way:

$$\frac{\partial a_i^*}{\partial q_i} > 0, \; \frac{\partial a_i^*}{\partial q_j} < 0, \tag{S13}$$

where $j \neq i$. To show this we note that equation (13) holds for all qualities, so we can take the partial derivative with respect to q_j, leading to

$$\frac{1}{g^2} B''(\bar{a}^*) \sum_{k=1}^{g} \frac{\partial a_k^*}{\partial q_j} = \frac{\partial^2 K(a_i^*, q_i)}{\partial a_i^2} \frac{\partial a_i^*}{\partial q_j} + \frac{\partial^2 K(a_i^*, q_i)}{\partial a_i \partial q_i} \delta_{ij}, \tag{S14}$$

for $i, j = 1, \ldots, g$. If we let A be the matrix with elements $A_{ij} = \partial a_i^* / \partial q_j$, we can write this as

$$(G + H)A = F, \tag{S15}$$

where G and F are diagonal matrices with

$$\begin{aligned} G_{ii} &= \frac{\partial^2 K(a_i^*, q_i)}{\partial a_i^2} > 0 \\ F_{ii} &= -\frac{\partial^2 K(a_i^*, q_i)}{\partial a_i \partial q_i} > 0, \end{aligned} \tag{S16}$$

and $H = \beta J$ where J is a matrix with all elements equal to one and

$$\beta = -\frac{1}{g^2} B''(\bar{a}^*) > 0. \tag{S17}$$

Noting that H has rank 1, we can use a result from [37] to write the inverse of $G + H$ as

$$(G + H)^{-1} = G^{-1} - \frac{1}{1+\gamma} G^{-1} H G^{-1}, \tag{S18}$$

where

$$\gamma = \mathrm{tr}(HG^{-1}) = \beta \sum_{i=1}^{g} \frac{1}{G_{ii}} > 0. \tag{S19}$$

The solution to equation (S15) is then

$$A = G^{-1}F - \frac{\beta}{1+\gamma} G^{-1} J G^{-1} F. \tag{S20}$$

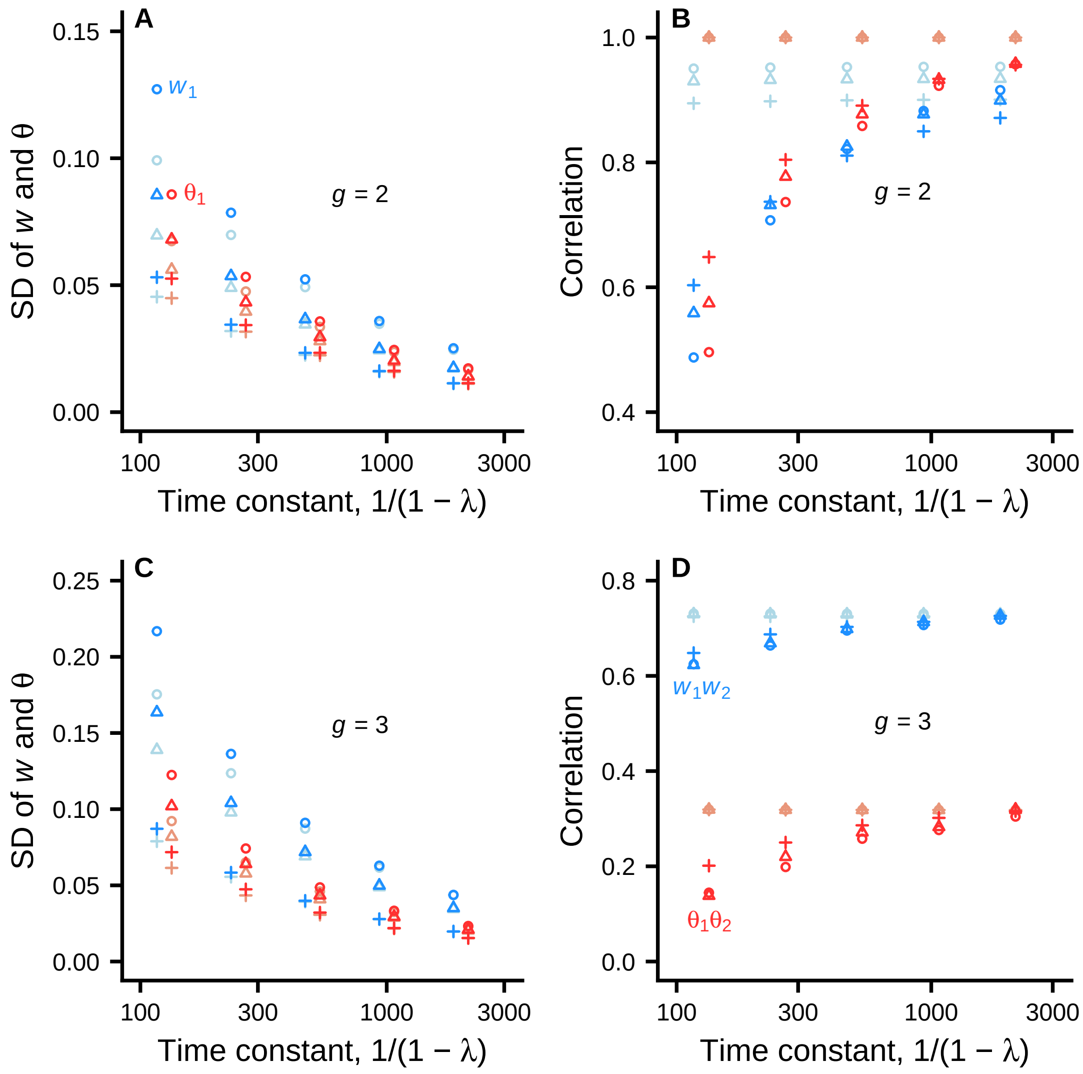

Figure S1. Illustration of how the limiting variance-covariance matrix from equations (S8, S12) is approached by the multivariate distribution of the learning parameters w_i and θ_i after many rounds of learning, for successively smaller rates of learning. The rate of learning is expressed through a time constant, $1/(1-\lambda)$, where λ is the leading eigenvalue of the matrix A from equations (S9, S10). Panel A shows the SD of w_1 (blue) and θ_1 (red) for $g = 2$, shifted slightly left and right to avoid overlap. The lighter coloured symbols are the limiting predictions from equation (S12) and the bolder symbols show values from individual-based simulations. Approach to the limit means that bold symbols come closer to the corresponding lighter coloured symbols. The different symbol shapes indicate different values of $\bar{q}$, for group compositions as in Fig. 2 ($\bar{q} = 0$, circle; $\bar{q} = 1/2$, triangle; $\bar{q} = 1$, plus). Panel B shows correlations between w_1 and w_2 (blue) and between θ_1 and θ_2 in the same way ($\bar{q} = 0$, circle; $\bar{q} = 1/3$, triangle; $\bar{q} = 1$, plus). Note that the limiting distribution is degenerate when $g = 2$, with the limiting correlation between θ_1 and θ_2 equal to 1. Panels C and D illustrate the same thing for $g = 3$.

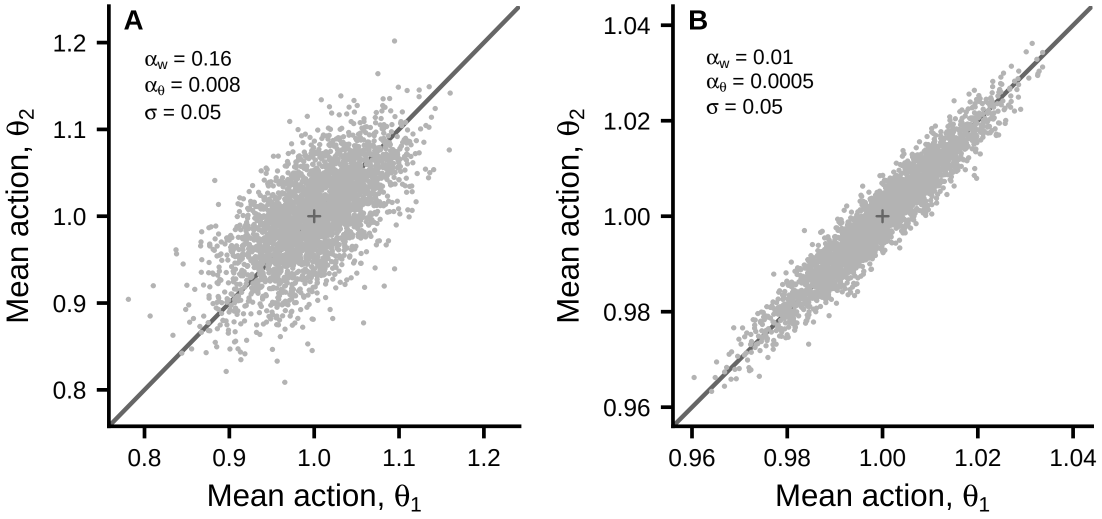

Figure S2. A snapshot of the the values of the mean actions θ_1 and θ_2 from simulations of many groups of size $g = 2$. The individual qualities in each group are $q_1 = q_2 = 1$. Panel A has the same parameters as the leftmost red plus symbol in Fig. S1B, which corresponds to the highest rates of learning, and panel B has the same parameters as the rightmost red plus symbol in Fig. S1B, which corresponds to the lowest rates of learning. In each panel the line, with slope 1 and intercept 0, shows the limiting relation of a correlation equal to 1. The plus symbol at $\theta_1 = \theta_2 = 1$ shows the location of the Nash equilibrium.

As $G^{-1}F$ is a diagonal matrix, we see directly that $\partial a_i^*/\partial q_j < 0$ for $j \neq i$, and then it follows from equation (S14) that $\partial a_i^*/\partial q_i > 0$.

For our special case of equations (2, 3), one readily solves equation (13), yielding equation (14) with

$$e_0 = \frac{B_1 - gK_1}{gK_{11} - B_2} \tag{S21}$$
$$e_1 = -\frac{K_{12}\left(g^2K_{11} - (g-1)B_2\right)}{gK_{11}(gK_{11} - B_2)}$$
$$e_2 = -\frac{B_2K_{12}}{gK_{11}(gK_{11} - B_2)}.$$

We note that $e_1 > 0$ and $e_2 < 0$, so that

$$\frac{\partial a_i^*}{\partial q_i} = e_1 > 0, \; \frac{\partial a_j^*}{\partial q_i} = e_2 < 0, \tag{S22}$$

with $j \neq i$, which agrees with equation (S13). Also, $g = 1$ is a special case where e_2 is not relevant, but e_0 and e_1 above apply to this case. The sensitivity of the equilibrium actions to differences in quality between group members can be written

$$a_i^* - a_j^* = -\frac{K_{12}}{K_{11}}(q_i - q_j). \tag{S23}$$

Evolution of cognitive bias

First, if the true qualities of group members are q_i, an evolutionary equilibrium for the perceived qualities p_i should satisfy

$$\frac{dW_i}{dp_i} = B'(\bar{a}^*(p.))\frac{\partial \bar{a}^*}{\partial p_i} - \frac{\partial K}{\partial a_i}(a_i^*(p.), q_i)\frac{\partial a_i^*(p.)}{\partial p_i} = 0, \tag{S24}$$

for $i = 1, \ldots, g$, where W_i is the Darwinian reproductive value from equation (4). Note that this expression for the derivative takes into account that a Nash equilibrium $a^*(p.)$ depends and the perceived qualities of the group members. Using equation (15), this becomes the condition for the derivative in equation (16). Next, replacing the true qualities q_i with the perceived qualities p_i, equation (14) for the Nash equilibrium becomes

$$a_i^*(p.) = e_0 + e_1 p_i + e_2 \sum_{j \neq i} p_j = e_0 + e_1 p_i + e_2(g-1)\bar{p}_{-i}. \tag{S25}$$

It follows that

$$\bar{a}^*(p.) = \frac{1}{g}\sum_{i=1}^{g} a_i^*(p.) = \frac{1}{g}\sum_{i=1}^{g}\big(e_0 + e_1 p_i + e_2(g-1)\bar{p}_{-i}\big) \\ = e_0 + \big(e_1 + (g-1)e_2\big)\bar{p} = e_0 + e_{12} g \bar{p}, \tag{S26}$$

where we introduced the notation

$$e_{12} = \frac{1}{g}\big(e_1 + (g-1)e_2\big) = -\frac{K_{12}}{gK_{11} - B_2}, \tag{S27}$$

and used equation (S21) for the right-hand side. With

$$\frac{\partial a_i^*(p.)}{\partial p_i} = e_1, \; \frac{\partial a_j^*(p.)}{\partial p_i} = e_2, \tag{S28}$$

for $j \neq i$, and using equation (16), equation (S24) becomes

$$K_{12}(p_i - q_i)e_1 + \frac{1}{g}\big(B_1 + B_2\bar{a}^*(p.)\big)(g-1)e_2 = 0.$$

This can be developed as

$$p_i - q_i = -\frac{(g-1)e_2}{gK_{12}e_1}\big(B_1 + B_2\bar{a}^*(p.)\big) \\ = -\frac{(g-1)B_2}{gK_{12}\big(g^2K_{11} - (g-1)B_2\big)}\big(B_1 + B_2(e_0 + e_{12}g\bar{p})\big) = \gamma_0 + \gamma_1\bar{p}, \tag{S29}$$

where we introduced the notation γ_0 and γ_1. Averaging over the group we get that $\bar{p} = (\gamma_0 + \bar{q})/(1-\gamma_1)$, so we can write

$$p_i - q_i = \beta_0 + \beta_1\bar{q},$$

with $\beta_0 = \gamma_0/(1-\gamma_1)$ and $\beta_1 = \gamma_1/(1-\gamma_1)$. This is then the evolutionary equilibrium in equation (17). After some tedious calculation we also get the expressions

$$\beta_0 = -\frac{(g-1)B_2(B_1K_{11} - B_2K_1)}{gK_{11}K_{12}\big(g^2K_{11} - (2g-1)B_2\big)} \\ \beta_1 = \frac{(g-1)B_2^2}{gK_{11}\big(g^2K_{11} - (2g-1)B_2\big)}. \tag{S30}$$

We can note that $\beta_0 < 0$ and $\beta_1 > 0$ for $g > 1$. Also, for large group size g the coefficients β_0 and β_1 approach 0, so that p_i approaches q_i as g becomes large.
